# Genkwanin mitigates renal fibrosis by reprogramming profibrotic signaling and restoring TFEB-mediated autophagy

**DOI:** 10.3389/fphar.2026.1861855

**Published:** 2026-08-13

**Authors:** Shen Wang, Yu He, Xin Gan, Hao Kang, Zhu Zhou, ZongHai He

**Affiliations:** 1 Department of Urology, Tongji Hospital of Tongji Medical College, Huazhong University of Science and Technology, Wuhan, China; 2 Hubei Provincial Clinical Medical Research Center for Minimally Invasive Treatment of Urology, Wuhan, China; 3 Department of Orthopedics, Tongji Hospital, Tongji Medical College, Huazhong University of Science and Technology, Wuhan, China; 4 Department of Nephrology, First Affiliated Hospital of Kunming Medical University, Kunming, China; 5 First Department of Urology, First Affiliated Hospital of Kunming Medical University, Kunming, China

**Keywords:** autophagy, genkwanin, LC3, renal fibrosis, TFEB

## Abstract

**Background:**

Renal fibrosis is a common pathological outcome of chronic kidney disease and is characterized by persistent profibrotic signaling and impaired cellular homeostasis. Genkwanin (GAK), a natural flavonoid compound, has shown anti-inflammatory and antioxidant activities in several disease models. However, its role in renal fibrosis remains unclear.

**Materials and methods:**

We investigated the antifibrotic effects of GAK in a TGF-β1–induced HK-2 cell model and a unilateral ureteral obstruction (UUO) mouse model. EMT- and fibrosis-related changes were assessed by RT-qPCR, Western blotting, immunofluorescence, and histological staining. The involvement of TGF-β1/SMAD2 and β-catenin signaling was examined using pharmacological modulators. Autophagic flux was evaluated by tandem mCherry-GFP-LC3 analysis, transmission electron microscopy, and autophagy-related protein expression. TFEB inhibition was used to further assess the role of lysosome–autophagy regulation.

**Results:**

GAK significantly attenuated TGF-β1–induced EMT and extracellular matrix accumulation in HK-2 cells and reduced renal fibrotic injury in UUO mice. Mechanistically, GAK decreased p-SMAD2 expression and limited β-catenin nuclear redistribution, indicating suppression of both TGF-β1/SMAD2 and β-catenin signaling. In addition, GAK improved autophagic flux and increased TFEB expression. Inhibition of TFEB partially weakened the protective effects of GAK on fibrosis-related and autophagy-related changes, suggesting that TFEB-mediated lysosome–autophagy function contributes to its antifibrotic action.

**Conclusion:**

In conclusion, GAK alleviates renal fibrosis *in vitro* and *in vivo*, at least in part, by suppressing TGF-β1/SMAD2 and β-catenin signaling and by restoring TFEB-associated autophagic flux.

## 1 Introduction

Renal fibrosis is a maladaptive, progressive scarring process characterized by tubulointerstitial injury, persistent inflammation, activation of myofibroblasts, and excessive extracellular matrix deposition, which collectively distort renal architecture and drive irreversible loss of kidney function (Huang et al., 2023; Nørregaard et al., 2023). It is widely regarded as a histopathological hallmark and final common pathway underpinning the progression of diverse etiologies of chronic kidney disease (CKD) toward kidney failure (Huang et al., 2023; Nørregaard et al., 2023; Yuan et al., 2025). A The global burden of CKD continues to rise, expanding the population at risk for renal fibrosis. The GBD 2023 analysis estimated that 788 million adults were living with CKD in 2023, with an age-standardized prevalence of 14.2% (Global and regional, 2025). Despite important advances in nephroprotective care, treatment remains constrained by a fundamental therapeutic gap. Current guideline recommended strategies, such as renin-angiotensin system inhibition, Sodium-Glucose Cotransporter 2 (SGLT2) inhibitors, and multifactorial risk control, primarily slow functional deterioration and reduce cardiorenal events but do not directly reverse established fibrotic remodeling, particularly once tubulointerstitial scarring is entrenched (Levin et al., 2024; Awdishu et al., 2025). Moreover, multiple antifibrotic approaches that were highly effective in preclinical models have shown limited clinical translation, reflecting disease heterogeneity, context-dependent signaling, and challenges in identifying actionable, kidney specific targets (Nørregaard et al., 2023; Hong et al., 2025; Park and Yoo, 2022).

Genkwanin (GAK) is an O-methylated flavone found in several medicinal plants. It is especially abundant in Daphne genkwa (Genkwa Flos), a traditional herbal drug rich in flavonoids (Kim et al., 2022; Kim et al., 2021; El Menyiy et al., 2023). GAK has been described as a plant-derived small-molecule scaffold with a relatively consistent pharmacological profile. Previous studies have reported its anti-inflammatory and antioxidant effects (El Menyiy et al., 2023; Fu et al., 2022). In innate immune cells, GAK suppresses LPS-induced inflammatory responses. It reduces iNOS expression and lowers key cytokine levels. Mechanistic studies also suggest that GAK inhibits NF-κB related signaling and the transcription of inflammatory mediators (Balendran et al., 2023). Similar pathway-level effects have been reported in rheumatoid arthritis-related models. In these systems, GAK decreases NF-κB activation, reduces iNOS-linked inflammatory tone, and limits the abnormal proliferation of fibroblast-like synoviocytes. GAK has also been reported to support redox balance and cellular protection. It lowers ROS levels in epithelial injury settings. It can also improve mitochondrial dysfunction. Some studies report that GAK activates the SIRT1–Nrf2/HO-1 antioxidant pathway (Chen et al., 2022; Liu et al., 2026). These changes are often accompanied by reduced apoptosis. Preclinical evidence further supports these activities in disease models. Several studies report benefits in DSS-induced colitis. Others show protection in paraquat-induced acute lung injury. GAK has also shown effects in allergic asthma models, including those linked to airway epithelial ferroptosis. In addition, cell-based Parkinson’s disease models suggest that GAK can suppress the TLR4/MyD88/NLRP3 inflammasome axis (Chen et al., 2022; Liu et al., 2026; Chen et al., 2025; Li et al., 2021a).

The TGF-β1/SMAD2 and β-catenin pathways are canonical profibrotic signaling axes that promote epithelial injury, EMT-like changes, and extracellular matrix accumulation during renal fibrosis and impaired TFEB-mediated autophagy-lysosome function contributes to tubular injury and renal fibrosis, whereas activation of mTORC1/TFEB-dependent lysosomal and autophagy programs can alleviate renal injury in experimental kidney disease models. Although many natural products have been reported to attenuate renal fibrosis by regulating TGF-β/SMAD, Wnt/β-catenin, or autophagy-related pathways, the role of GAK in renal fibrogenesis has not been defined. Therefore, the aiming of this study is to identify the antifibrotic effect of GAK on renal fibrosis, and the major novelty of this study is the evaluation of GAK in experimental renal fibrosis and the linkage of its antifibrotic effect to TFEB-related autophagic flux.

## 2 Methods and materials

### 2.1 Chemicals and reagents

GAK (#HY-N0731), lithium chloride (LiCl, #HY-Y0649), bleomycin hydrochloride (BLM, #HY-17565 A), Bafilomycin A1 (Baf, # HY-100558) and Eltrombopag (ETB, #HY-15306) were purchased from MedChemExpress (New Jersey, United States). TGF-β1 was purchased from Sigma-Aldrich (Massachusetts, United States). Primary antibodies against F4/80 (#29414-1-AP), E-cadherin (#20874-1-AP), N-cadherin (#22018-1-AP), collagen α type 1 (Col α1, #67288-1-Ig), vimentin (#10366-1-AP), ATG7 (#10088-2-AP), ATG5 (#10181-2-AP), ATG3 (#11262-2-AP), TFEB (#13372-1-AP), LC3 (#14600-1-AP), and Beclin1 (#11306-1-AP) were obtained from Proteintech (Wuhan, China). Primary antibodies against α-SMA (#A17910), p-SMAD2 (#AP1342), SMAD2 (#A19114), GAPDH (#A19056), β-catenin (#A19657), Phospho-β-Catenin-S552 (P-β-Catenin, #AP1315), anti-Non-phospho (Active) β-Catenin S33/S37/T41 (A-β-Catenin, #A22180), and P62 (#A19700) were purchased from ABclonal (Wuhan, China). Anti-TGF-β1 (#A15103) were purchased form Abcam (Cambridge, United Kingdom), HRP-conjugated secondary antibodies against rabbit and mouse IgG (#SA00001–2 and #SA00001–1) for Western blotting were obtained from Proteintech (Wuhan, China). Cy3-conjugated goat anti-rabbit IgG (H + L) (#AS007) and Cy3-conjugated goat anti-mouse IgG (H + L) (#AS008) were purchased from ABclonal (Wuhan, China).

### 2.2 Cell culture

Human proximal tubule epithelial cell line (HK-2) cells were cultured at 37 °C in a humidified incubator with 5% CO_2_. Cells were maintained in DMEM/F12 medium (#PYG0004-6, Boster, Wuhan, China) supplemented with 10% fetal bovine serum (FBS; #A5256701, Gibco, Waltham, United States). To induce fibrotic changes, HK-2 cells were treated with TGF-β1 (10 ng/mL) for 48 h. Before certain treatments, cells were serum starved for 24 h in medium containing 0.5% FBS.

### 2.3 Cytotoxicity assay

A Cell Counting Kit-8 (CCK-8) kit (#BS350B, Biosharp Life Sciences, Beijing, China) was used to assess the cytotoxicity of GAK in HK-2 cells. Cells were seeded into 96-well plates at a density of 1 × 10^4^ cells per well. After 24 h, cells were treated with different concentrations of GAK (2.5, 5, 10, 20, 50, 80 μM) for another 24 h. The medium was then replaced, and 90 μL of fresh medium plus 10 μL of CCK-8 reagent was added to each well. Plates were incubated in the dark for 1 h and absorbance was measured at 450 nm using a microplate reader (Thermo Fisher Scientific, Waltham, United States).

### 2.4 Histology, immunohistochemical and immunofluorescence staining

H&E staining was used to assess renal morphology, and collagen deposition/fibrosis was evaluated by Masson’s trichrome staining. For immunohistochemistry, sections were incubated with primary antibodies against Col α1, F4/80, TFEB and α-SMA overnight at 4 °C and Sections were then incubated with secondary antibodies and counterstained with hematoxylin. Images were captured using a Leica microscope (#DMI6000B, Germany). For immunofluorescence, cells were grown on coverslips and fixed. After blocking with 5% bovine serum albumin (BSA), cells were incubated with primary antibodies against fibronectin (1:100), TGF-β1 (1:100), β-catenin (1:100), P62 (1:100), and LC3 (1:100). Cells were then incubated with Cy3-conjugated secondary antibodies and Nuclei were stained with DAPI, and coverslips were mounted. Fluorescence images were acquired using an Olympus microscope (#BX63, Japan).

### 2.5 Quantitative real-time PCR (RT-qPCR)

Total RNA was extracted from HK-2 cells using Trizol reagent (#15596-026CN, Thermo Fisher Scientific, Massachusetts, United States), and RNA concentration was measured with a NanoDrop 2000 (Thermo Fisher Scientific, Massachusetts, United States). cDNA was synthesized using a reverse transcription kit (#MR101–01, Vazyme Biotech, Nanjing, China) according to the manufacturer’s instructions. RT-qPCR was performed using an RT-qPCR kit (#Q113–02, Vazyme Biotech, Nanjing, China) and relative expression was calculated using the 2^−ΔΔCT^ method. Primer sequences are listed in Table 1.

**TABLE 1:** Oligonucleotide primer sequences used for polymerase chain reaction amplification.

Gene	N (Forward, 5’→3′)	R (Reverse, 5’→3′)
E-cadherin (H)	GCC​TCC​TGA​AAA​GAG​AGT​GGA​AG	TGG​CAG​TGT​CTC​TCC​AAA​TCC​G
N-cadherin (H)	CCT​CCA​GAG​TTT​ACT​GCC​ATG​AC	GTA​GGA​TCT​CCG​CCA​CTG​ATT​C
Col1 (H)	GAT​TCC​CTG​GAC​CTA​AAG​GTG​C	AGC​CTC​TCC​ATC​TTT​GCC​AGC​A
Vimentin (H)	AGG​CAA​AGC​AGG​AGT​CCA​CTG​A	ATC​TGG​CGT​TCC​AGG​GAC​TCA​T
α-SMA (H)	CTA​TGC​CTC​TGG​ACG​CAC​AAC​T	CAG​ATC​CAG​ACG​CAT​GAT​GGC​A
P62 (H)	TGT​GTA​GCG​TCT​GCG​AGG​GAA​A	AGT​GTC​CGT​GTT​TCA​CCT​TCC​G
Beclin-1 (H)	CTG​GAC​ACT​CAG​CTC​AAC​GTC​A	CTC​TAG​TGC​CAG​CTC​CTT​TAG​C
ATG7 (H)	CGT​TGC​CCA​CAG​CAT​CAT​CTT​C	CAC​TGA​GGT​TCA​CCA​TCC​TTG​G
ATG5 (H)	GCA​GAT​GGA​CAG​TTG​CAC​ACA​C	GAG​GTG​TTT​CCA​ACA​TTG​GCT​CA
ATG3 (H)	ACT​GAT​GCT​GGC​GGT​GAA​GAT​G	GTG​CTC​AAC​TGT​TAA​AGG​CTG​CC
TFEB (H)	CCT​GGA​GAT​GAC​CAA​CAA​GCA​G	TAG​GCA​GCT​CCT​GCT​TCA​CCA​C
E-cadherin (M)	GGT​CAT​CAG​TGT​GCT​CAC​CTC​T	GCT​GTT​GTG​CTC​AAG​CCT​TCA​C
N-cadherin (M)	CCT​CCA​GAG​TTT​ACT​GCC​ATG​AC	CCA​CCA​CTG​ATT​CTG​TAT​GCC​G
Col1 (M)	CCT​CAG​GGT​ATT​GCT​GGA​CAA​C	CAG​AAG​GAC​CTT​GTT​TGC​CAG​G
Vimentin (M)	CGG​AAA​GTG​GAA​TCC​TTG​CAG​G	AGC​AGT​GAG​GTC​AGG​CTT​GGA​A
α-SMA (M)	TGC​TGA​CAG​AGG​CAC​CAC​TGA​A	CAG​TTG​TAC​GTC​CAG​AGG​CAT​AG
P62 (M)	GCT​CTT​CGG​AAG​TCA​GCA​AAC​C	GCA​GTT​TCC​CGA​CTC​CAT​CTG​T
Beclin-1 (M)	CAG​CCT​CTG​AAA​CTG​GAC​ACG​A	CTC​TCC​TGA​GTT​AGC​CTC​TTC​C
Atg7 (M)	CCT​GTG​AGC​TTG​GAT​CAA​AGG​C	GAG​CAA​GGA​GAC​CAG​AAC​AGT​G
Atg5 (M)	CTT​GCA​TCA​AGT​TCA​GCT​CTT​CC	AAG​TGA​GCC​TCA​ACC​GCA​TCC​T
Atg3 (M)	TAA​GGC​TGA​CGC​TGG​AGG​TGA​A	GTG​CTC​AAC​TGT​TAA​AGG​CTG​CC
Tfeb (M)	CGC​CTG​GAG​ATG​ACT​AAC​AAG​C	GGC​AAC​TCT​TGC​TTC​ACC​ACC​T

### 2.6 Western blot

Total protein was extracted using RIPA lysis buffer (#AR0102, Boster, Wuhan, China), and protein concentration was determined using a BCA assay. Proteins were separated on a 10% sodium dodecyl sulfate–polyacrylamide gel (SDS-PAGE), and then transferred onto polyvinylidene difluoride (PVDF) membranes (#IPVH00010, Millipore, Boston, United States). The membranes were blocked with 5% defatted milk powder for 1 h and after blocking, membranes were incubated with primary antibodies overnight at 4 °C. On the next day, membranes were incubated with secondary antibodies at 25 °C for 1 h, then protein signals were developed using enhanced chemiluminescence (ECL) reagent (#BMU102, Abbkine, Wuhan, China), and band intensity was recorded using a Bio-Rad imaging scanner (Bio-Rad, United States).

### 2.7 GFP-RFP-LC3 adenovirus transfection

To detect autophagic flux intensity, HK-2 cells were seeded into confocal dishes and then the cells were incubated with TGF-β1 and GAK (50 μM) for 48 h. After incubation, the cells were transfected with lentiviruses GFP-mCherry-LC3 (#C3011, Beyotime, Shanghai, China) for 24 h.

### 2.8 Transmission electron microscopy (TEM) and confocal microscopy

For TEM analysis, HK-2 cells treated with TGF-β1 and GAK were fixed in 2.5% glutaraldehyde in 0.1 M phosphate buffer, post-fixed in 1% osmium tetroxide, dehydrated through a graded ethanol series, and embedded in epoxy resin. Ultrathin sections were stained with uranyl acetate and lead citrate and samples were examined by TEM at 80 kV (Hitachi, Japan). For cells transduced with GFP-mCherry-LC3 lentivirus, fluorescence images were acquired using a laser scanning confocal microscope (Zeiss, Germany).

### 2.9 siRNA-mediated TFEB knockdown

To determine whether TFEB is functionally involved in the protective effect of GAK, TFEB expression was silenced in HK-2 cells using small interfering RNA (siRNA). HK-2 cells were seeded in 6-well plates and transfected when they reached approximately 60%–70% confluence. TFEB-specific siRNA (siTFEB; sense: 5′-GAA​AGG​AGA​CGA​AGG​UUC​AAC​AUC​A-3′; siTFEB antisense: 5′-UGA​UGU​UGA​ACC​UUC​GUC​UCC​UUU​C-3′) or negative control siRNA (si-NC) was transfected using Lipofectamine 3,000 transfection reagent (Invitrogen, United States) according to the manufacturer’s instructions. The final siRNA concentration was 50 nM. After [24 h] of transfection, cells were serum-starved in medium containing 0.5% FBS for 24 h and then treated with TGF-β1 (10 ng/mL) in the presence or absence of GAK (50 μM) for 48 h. Cells were subsequently collected for Western blotting analysis. The knockdown efficiency of TFEB was confirmed by detecting TFEB protein expression.

### 2.10 Animal experiments

Male C57BL/6 mice (20–25 g, 6–8 weeks old) were obtained from the Hubei Provincial Centers for Disease Control and Prevention (Wuhan, China). Mice were housed under specific pathogen-free conditions with controlled temperature, humidity, and a 12 h light/dark cycle, with free access to food and water. After acclimatization for 4 days, mice were randomly assigned using 15 to three groups: sham surgery (n = 5), UUO (n = 5), and UUO + GAK (n = 5). The UUO model was established by unilateral ureteral ligation according to established protocols. Mice in the UUO + GAK group received GAK at 20 mg/kg body weight via oral gavage once daily for 14 days, whereas mice in the sham and UUO groups received an equal volume of vehicle. Postoperative analgesia was provided using buprenorphine 0.1 mg/kg every 8 h for 3 days subcutaneously according to the approved animal protocol. All mice were euthanized on day 14, and blood and kidney tissues were collected for biochemical and histological analyses. All animal procedures were approved by the Animal Care and Use Committee of Tongji Medical College, Huazhong University of Science and Technology (No. TJ-C20210145) and were conducted in accordance with ARRIVE 2.0 guidelines.

### 2.11 Measurement of kidney function

The blood urea nitrogen (BUN) and serum creatinine (CR) was tested using Urea Assay Kit (#C013-2–1, Jiancheng China) and Creatinine Assay kit (#C011-2–1, Jiancheng China) according to the manufacturer’s instructions.

### 2.12 Statistical analysis

For *in vitro* experiments, all biological experiments were performed at least three times and all data are presented as mean ± SD. Normality was assessed using the Shapiro-Wilk test, and homogeneity of variance was evaluated using Levene’s test. For comparisons between two groups, normally distributed data with equal variances were analyzed using an unpaired two-tailed Student’s t-test while for comparisons among multiple groups, one-way or two-way ANOVA followed by Tukey’s test was performed as appropriate. Statistical analyses were performed using GraphPad Prism version 9.5. P < 0.05 was considered statistically significant. Significance is indicated as *P < 0.05 and **P < 0.01.

## 3 Results

### 3.1 GAK has no viability inhibition for HK-2 cells

**FIGURE 1:**
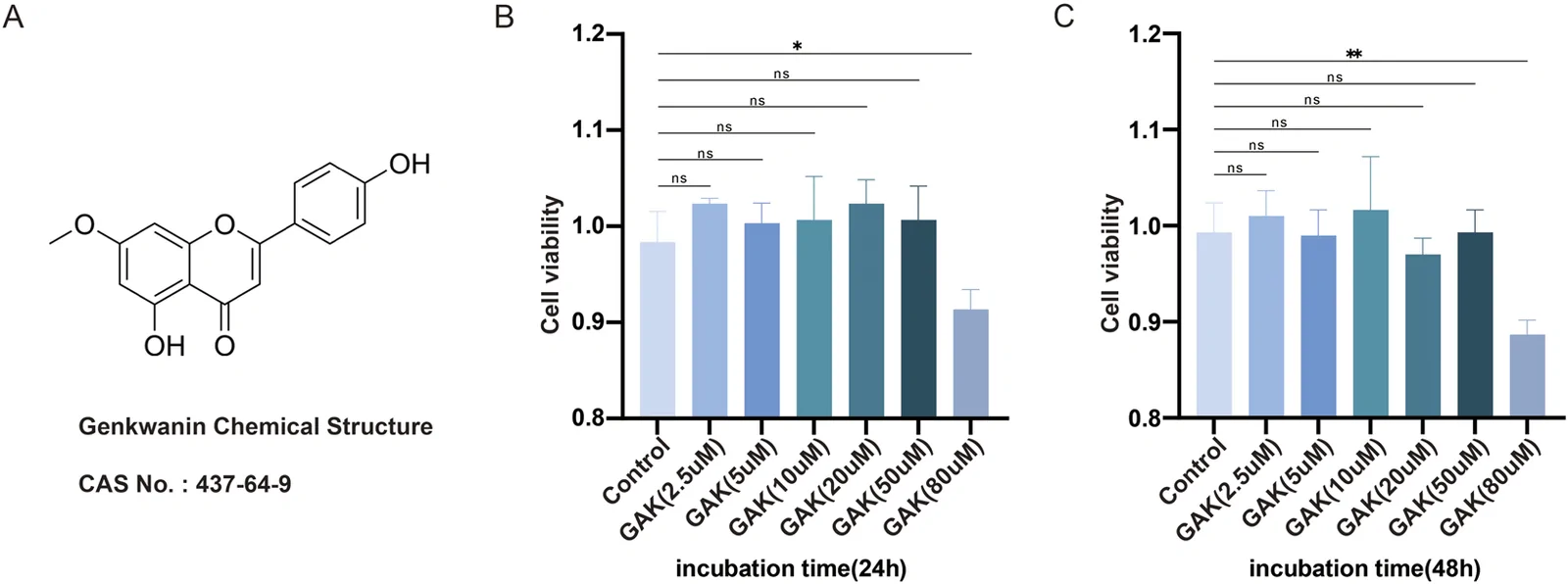
Chemical structure of genkwanin and cytotoxicity assessment in HK-2 cells. **(A)** Chemical structure of genkwanin (GAK). **(B,C)** HK-2 cells were treated with increasing concentrations of GAK (2.5, 5, 10, 20, 50, and 80 μM) for 24 h **(B)** or 48 h **(C)**, and cell viability was assessed using the CCK-8 assay. Vehicle-treated cells served as the control group. Data are presented as mean ± SD. Statistical significance was determined by one-way ANOVA followed by Dunnett’s multiple-comparisons test versus the control group. Ns, not significant; *P < 0.05; **P < 0.01.

We first assessed the cytotoxicity of GAK in HK-2 cells using a CCK-8 assay. The chemical structure of GAK is shown in Figure 1A. As shown in Figures 1B,C, GAK did not significantly reduce cell viability at concentrations up to 50 μM at either time point. In contrast, 80 μM caused a mild decrease in viability. These results indicate that GAK has low cytotoxicity within the working range and support using ≤50 μM for subsequent *in vitro* experiments.

### 3.2 GAK alleviates epithelial mesenchymal transition (EMT) in TGF-β1 induced HK-2 cells

**FIGURE 2:**
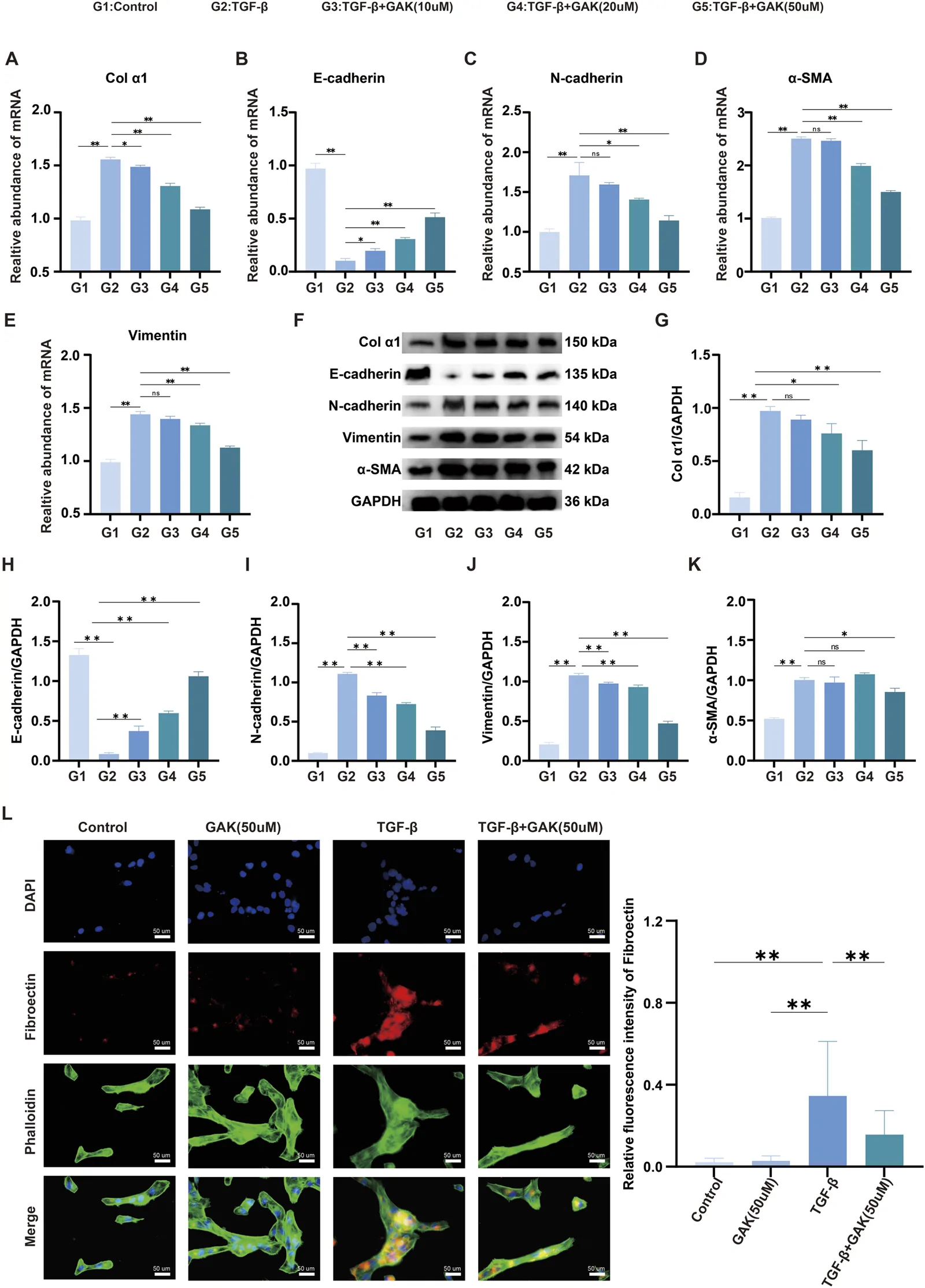
GAK attenuates TGF-β1-induced EMT and extracellular matrix accumulation in HK-2 cells. HK-2 cells were stimulated with TGF-β1 (10 ng/mL, 48 h) in the presence or absence of GAK (10, 20, or 50 μM). **(A–E)** RT-qPCR analysis of Col α1 **(A)**, E-cadherin **(B)**, N-cadherin **(C)**, α-SMA **(D)**, and vimentin **(E)** mRNA expression. **(F)** Representative Western blot images of EMT- and fibrosis-related proteins, including E-cadherin, N-cadherin, Col α1, vimentin, and α-SMA, with GAPDH as the loading control. **(G–K)** Densitometric quantification of E-cadherin/GAPDH **(G)**, N-cadherin/GAPDH **(H)**, Col α1/GAPDH **(I)**, vimentin/GAPDH **(J)**, and α-SMA/GAPDH **(K)**. **(L)** Representative immunofluorescence images of fibronectin (red), phalloidin-labeled cytoskeleton (green), and DAPI-stained nuclei (blue). Scale bars are indicated in the images. (M) Quantification of fibronectin fluorescence intensity. Data are presented as mean ± SD. Statistical significance was determined by one-way ANOVA followed by Tukey’s multiple-comparisons test. Ns, not significant; *P < 0.05; **P < 0.01.

EMT is widely recognized as a key process in renal fibrosis. Based on previous studies, we treated HK-2 cells with TGF-β1 (10 ng/mL) for 48 h to induce EMT and model a profibrotic response. We then examined how GAK affects TGF-β1–induced EMT in HK-2 cells. RT-qPCR results showed that TGF-β1 significantly increased the mRNA levels of fibrotic markers (Col α1 and α-SMA) and mesenchymal markers (N-cadherin and vimentin). TGF-β1 also significantly reduced the mRNA level of the epithelial marker E-cadherin (Figures 2A–E). GAK treatment reduced these TGF-β1 induced changes in a dose-dependent manner at 10, 20, and 50 μM. Specifically, GAK decreased Col α1, α-SMA, N-cadherin, and vimentin, and it restored E-cadherin compared with the TGF-β1 group. Western blot analysis further confirmed the same pattern at the protein level, and the protein changes also showed a dose-dependent trend (Figures 2F–K). In addition, we performed immunofluorescence staining to evaluate extracellular matrix (ECM) accumulation. TGF-β1 markedly increased fibronectin fluorescence intensity in HK-2 cells, while GAK clearly reduced this increase (Figures 2L,M).

### 3.3 GAK inhibits EMT by negatively regulating the Wnt/β-catenin and TGF-β1/SMAD2 signaling pathways in TGF-β1 induced HK-2 cells

Previous studies have shown that the Wnt/β-catenin and TGF-β1/SMAD2 pathways are closely linked to the development of renal fibrosis (Gu et al., 2023; Li et al., 2026; Li et al., 2021b). These pathways are often activated under fibrotic conditions. Many reports also suggest that blocking either pathway can protect tubular epithelial cells and slow fibrosis progression. Based on this background, we examined whether GAK modulates these two pathways in TGF-β1–stimulated HK-2 cells, and we further tested whether pathway activation could weaken the protective effects of GAK.

**FIGURE 3:**
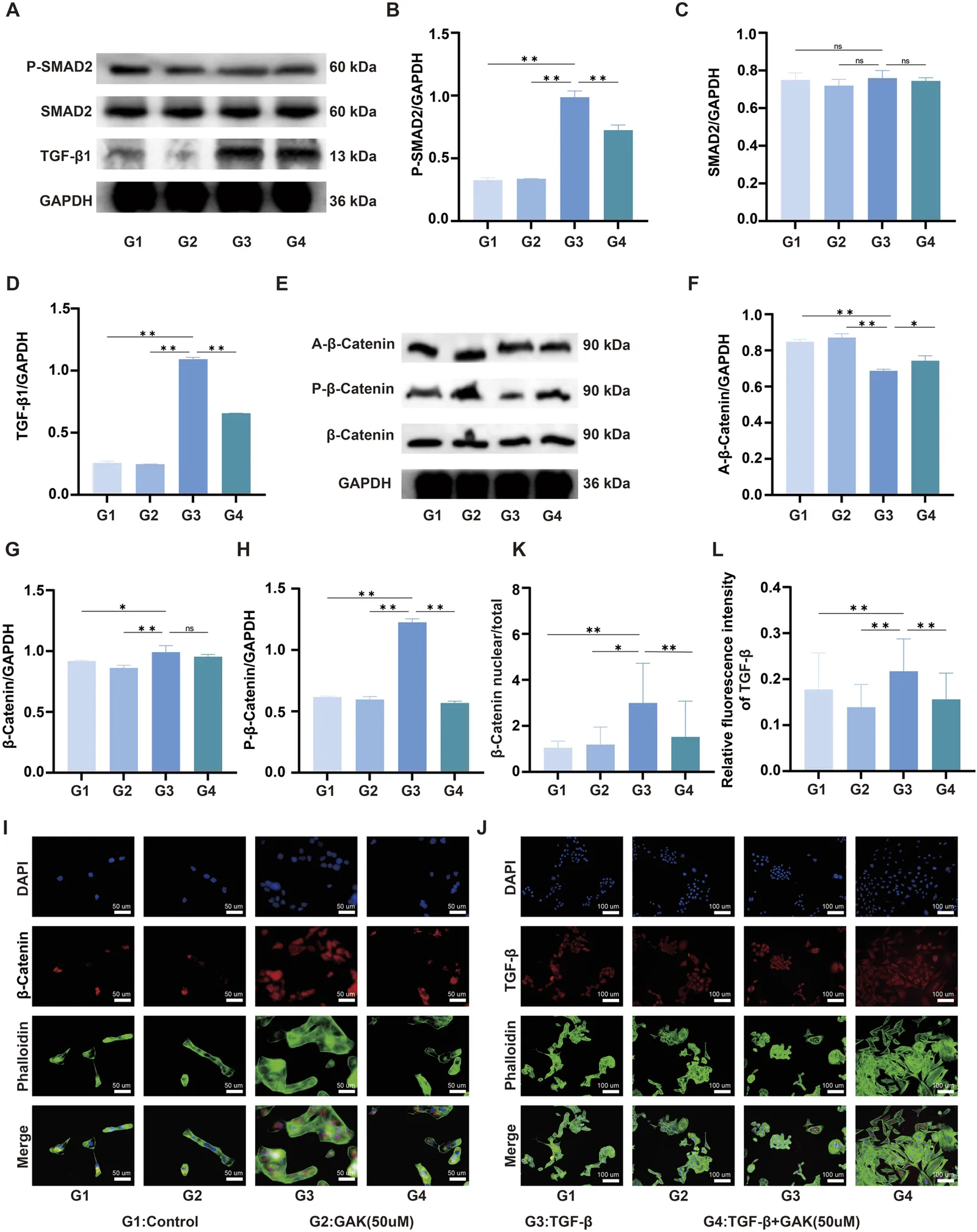
GAK suppresses TGF-β1/SMAD2 and β-catenin signaling activation in TGF-β1-stimulated HK-2 cells. HK-2 cells were treated with TGF-β1 (10 ng/mL, 48 h) with or without GAK (50 μM). **(A)** Representative Western blot images of p-SMAD2, total SMAD2, and TGF-β1, with GAPDH as the loading control. **(B–D)** Densitometric quantification of p-SMAD2/GAPDH **(B)**, SMAD2/GAPDH **(C)**, and TGF-β1/GAPDH **(D)**. **(E)** Representative Western blot images of active β-catenin, phospho-β-catenin, and total β-catenin. **(F–H)** Densitometric quantification of active β-catenin/GAPDH **(F)**, total β-catenin/GAPDH **(G)**, and phospho-β-catenin/GAPDH **(H)**. **(I)** Representative immunofluorescence images showing β-catenin localization. **(J)** Representative immunofluorescence images showing TGF-β1 expression. β-catenin or TGF-β1 is shown in red, phalloidin in green, and DAPI-stained nuclei in blue. Scale bars are indicated in the images. **(K)** Quantification of the nuclear/total β-catenin fluorescence ratio. **(L)** Quantification of TGF-β1 fluorescence intensity. Data are presented as mean ± SD. Statistical significance was determined by one-way ANOVA followed by Tukey’s multiple-comparisons test. Ns, not significant; *P < 0.05; **P < 0.01.

First, we assessed pathway activation by Western blotting. TGF-β1 stimulation increased Wnt/β-catenin signaling, as shown by lower levels of A-β-catenin, higher P-β-catenin, and total β-catenin. TGF-β1 also activated the TGF-β1/SMAD2 pathway, as shown by increased TGF-β1, SMAD2, and p-SMAD2 levels. GAK treatment reduced these TGF-β1–induced increases in both pathways (Figures 3A–H). We then used immunofluorescence to provide spatial support for these biochemical changes. In TGF-β1–treated HK-2 cells, β-catenin showed enhanced nuclear localization, and TGF-β1 staining intensity was increased. GAK reduced β-catenin nuclear translocation and lowered the TGF-β1 signal compared with the TGF-β1 group (Figures 3I–L).

**FIGURE 4:**
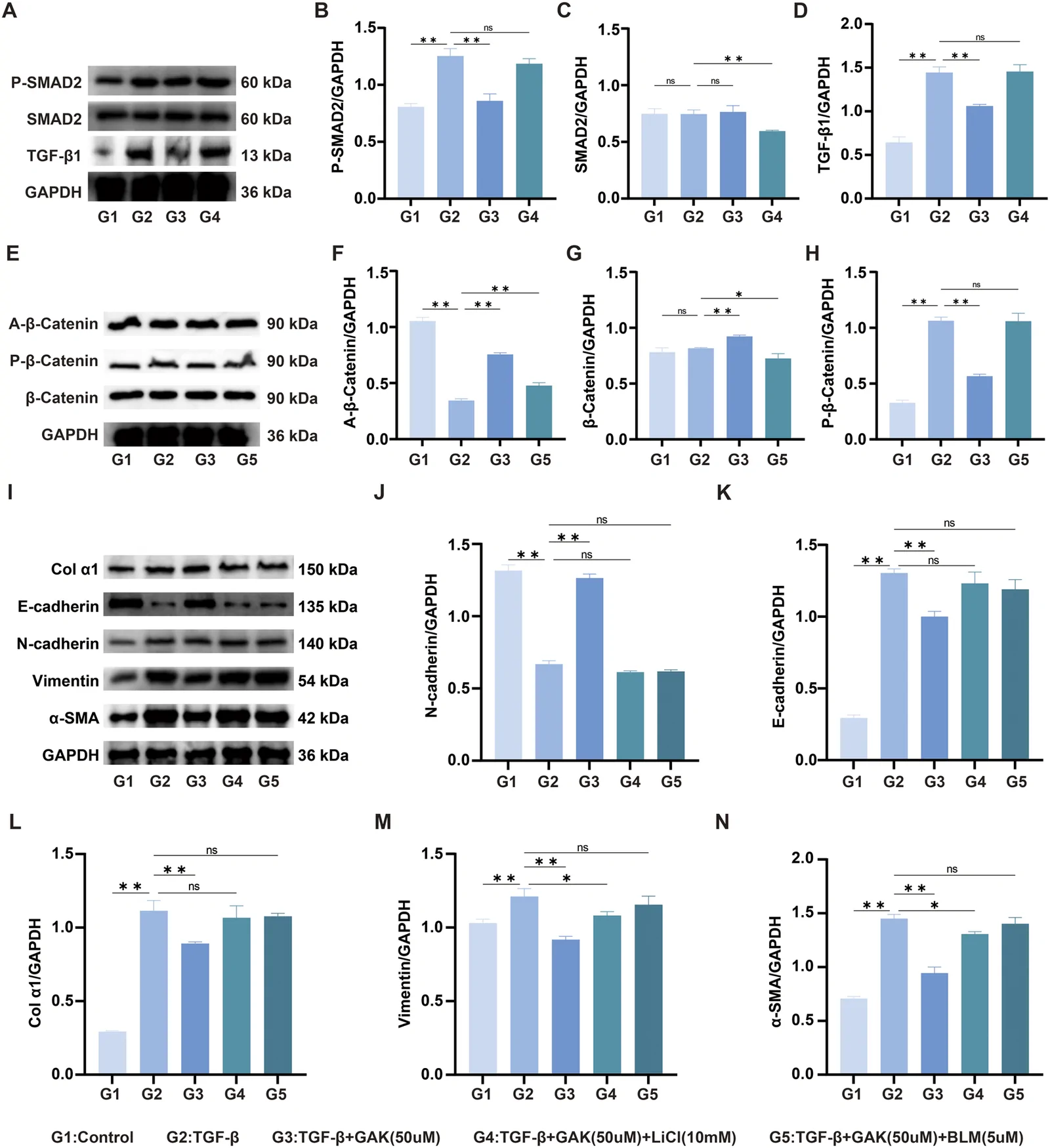
Pharmacological pathway perturbation supports the involvement of SMAD2 and β-catenin signaling in GAK-mediated protection. HK-2 cells were stimulated with TGF-β1 and treated with GAK in the presence or absence of pathway modulators, including LiCl and/or bleomycin (BLM), as indicated. Please insert the exact concentrations and treatment durations for LiCl and BLM according to the Methods section. **(A)** Representative Western blot images of p-SMAD2, total SMAD2, and TGF-β1, with GAPDH as the loading control. **(B–D)** Densitometric quantification of p-SMAD2/GAPDH **(B)**, SMAD2/GAPDH **(C)**, and TGF-β1/GAPDH **(D)**. **(E)** Representative Western blot images of active β-catenin, phospho-β-catenin, and total β-catenin. **(F–H)** Densitometric quantification of active β-catenin/GAPDH **(F)**, total β-catenin/GAPDH **(G)**, and phospho-β-catenin/GAPDH **(H)**. **(I)** Representative Western blot images of EMT- and fibrosis-related proteins, including E-cadherin, N-cadherin, Col α1, vimentin, and α-SMA. **(J–N)** Densitometric quantification of N-cadherin/GAPDH **(J)**, E-cadherin/GAPDH **(K)**, Col α1/GAPDH **(L)**, vimentin/GAPDH **(M)**, and α-SMA/GAPDH **(N)**. Data are presented as mean ± SD. Statistical significance was determined by one-way ANOVA followed by Tukey’s multiple-comparisons test. Ns, not significant; *P < 0.05; **P < 0.01.

**FIGURE 5:**
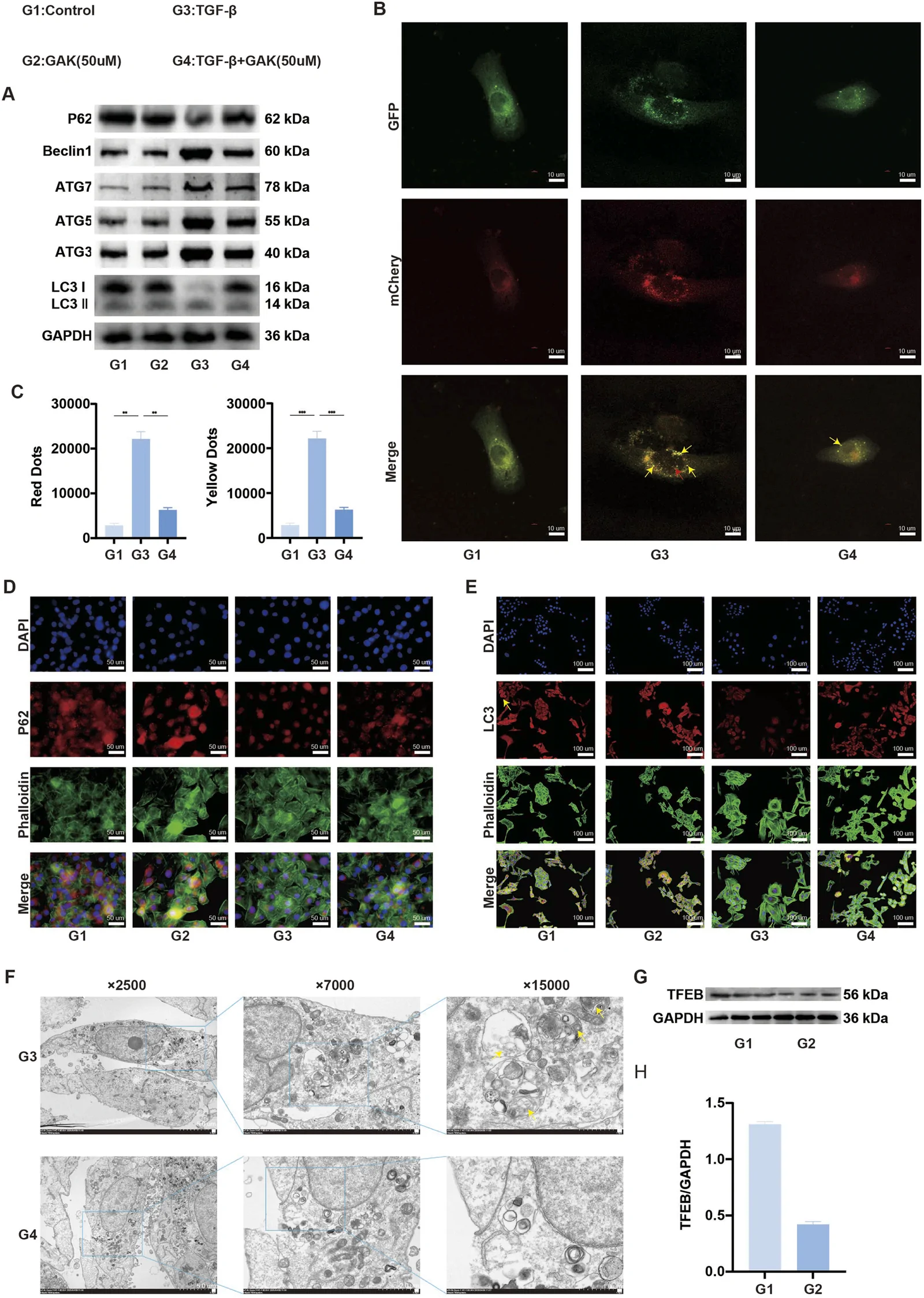
GAK restores autophagy homeostasis and autophagic flux in TGF-β1-stimulated HK-2 cells. HK-2 cells were treated with TGF-β1 (10 ng/mL, 48 h) in the presence or absence of GAK (50 μM). **(A)** Representative Western blot images of autophagy-related proteins, including P62, Beclin 1, ATG7, ATG5, ATG3, and LC3, with GAPDH as the loading control. **(B)** Representative confocal images from the tandem fluorescent mCherry-GFP-LC3 reporter assay. Yellow puncta indicate autophagosomes, whereas red-only puncta indicate autolysosomes. **(C)** Quantification of yellow and red-only LC3 puncta per cell. **(D,E)** Representative immunofluorescence images of P62 **(D)** and LC3 **(E)**, shown in red, with phalloidin in green and DAPI-stained nuclei in blue. Scale bars are indicated in the images. **(F)** Representative transmission electron microscopy images showing autophagy-related ultrastructural vesicles in the indicated groups. Yellow arrows indicate autophagosomes while ren arrows indicate autolysosomes, and scale bars are indicated in the images. **(G,H)** Representative Western blot images **(G)** and quantification **(H)** of TFEB expression in control and TGF-β1-treated HK-2 cells. Data are presented as mean ± SD. Statistical significance was determined by one-way ANOVA followed by Tukey’s multiple-comparisons test, except for two-group comparisons where an unpaired two-tailed Student’s t-test was used. Ns, not significant; *P < 0.05; **P < 0.01.

Next, we performed pharmacological rescue experiments to test pathway specificity. We treated TGF-β1–stimulated HK-2 cells with GAK together with LiCl, which activates β-catenin signaling, or with BLM, which as an additional pro-fibrotic stimulus that reactivates TGF-β/SMAD readouts in our setting. LiCl partially counteracted the inhibitory effect of GAK on β-catenin signaling, as shown by the recovery of β-catenin-related readouts (Figures 4A–D). Similarly, BLM partially counteracted the inhibitory effect of GAK on the TGF-β1/SMAD2 pathway (Figures 5E–H). Finally, we examined whether these pathway activators also affected the antifibrotic phenotype. Both LiCl and BLM weakened the ability of GAK to suppress EMT and fibrotic markers, leading to higher levels of EMT and fibrotic proteins compared with GAK treatment alone (Figures 4I–N).

### 3.4 GAK ameliorates TGF-β1-induced autophagy dysregulation and improves autophagic flux

Given the close relationship between autophagy and renal fibrosis (Dai et al., 2022; Ruby et al., 2023), we next investigated whether GAK affects autophagy in TGF-β1 stimulated HK-2 cells. RT-qPCR analysis of autophagy related genes showed that TGF-β1 induced an abnormal expression pattern of P62, Beclin1, ATG3, ATG5, and ATG7, while GAK partly normalized these changes, which suggests that GAK helps restore autophagy balance (Supplementary Figure S1A–E). Western blot analysis showed a similar trend at the protein level (Figure 5A; Supplementary Figure S2). To further evaluate autophagic flux, we performed tandem fluorescent mCherry-GFP-LC3 analysis. TGF-β1 altered the distribution of red and yellow LC3 puncta, indicating disrupted autophagic dynamics. GAK changed the puncta pattern and reduced abnormal puncta accumulation under TGF-β1 stimulation (Figures 5B,C). This interpretation was further supported by immunofluorescence staining of P62 and LC3, which showed that TGF-β1 caused abnormal P62 and LC3 signal accumulation, while GAK reduced these signals (Figures 5D,E; Supplementary Figure S3). TEM also showed ultrastructural differences in autophagic vesicles between the TGF-β1 and TGF-β1 + GAK groups, which is consistent with improved autophagic processing after GAK treatment (Figure 5F).

To further determine whether lysosome-dependent autophagic flux contributes to the protective effect of GAK, we used bafilomycin A1, a lysosomal inhibitor that blocks autophagosome degradation. After lysosomal degradation was blocked by bafilomycin A1, LC3B-II and P62 accumulated, confirming effective inhibition of autophagic degradation. Under this condition, the inhibitory effects of GAK on Col α1, N-cadherin, vimentin, and α-SMA were weakened, and E-cadherin expression was reduced compared with the TGF-β1 + GAK group (Supplementary Figure S4A).

We next used TFEB knockdown to further examine whether TFEB is functionally required for the effects of GAK. TFEB siRNA effectively reduced TFEB protein expression in TGF-β1-stimulated HK-2 cells. Compared with the TGF-β1 + GAK group, TFEB knockdown attenuated the ability of GAK to reduce Col α1, N-cadherin, vimentin, and α-SMA, and also weakened the restoration of E-cadherin expression. In addition, TFEB knockdown altered the effects of GAK on P62 and LC3B-I/II, indicating disruption of TFEB-related autophagy regulation (Supplementary Figure S4B).

### 3.5 ETB, a TFEB inhibitor, counteracts the protective effect of GAK

**FIGURE 6:**
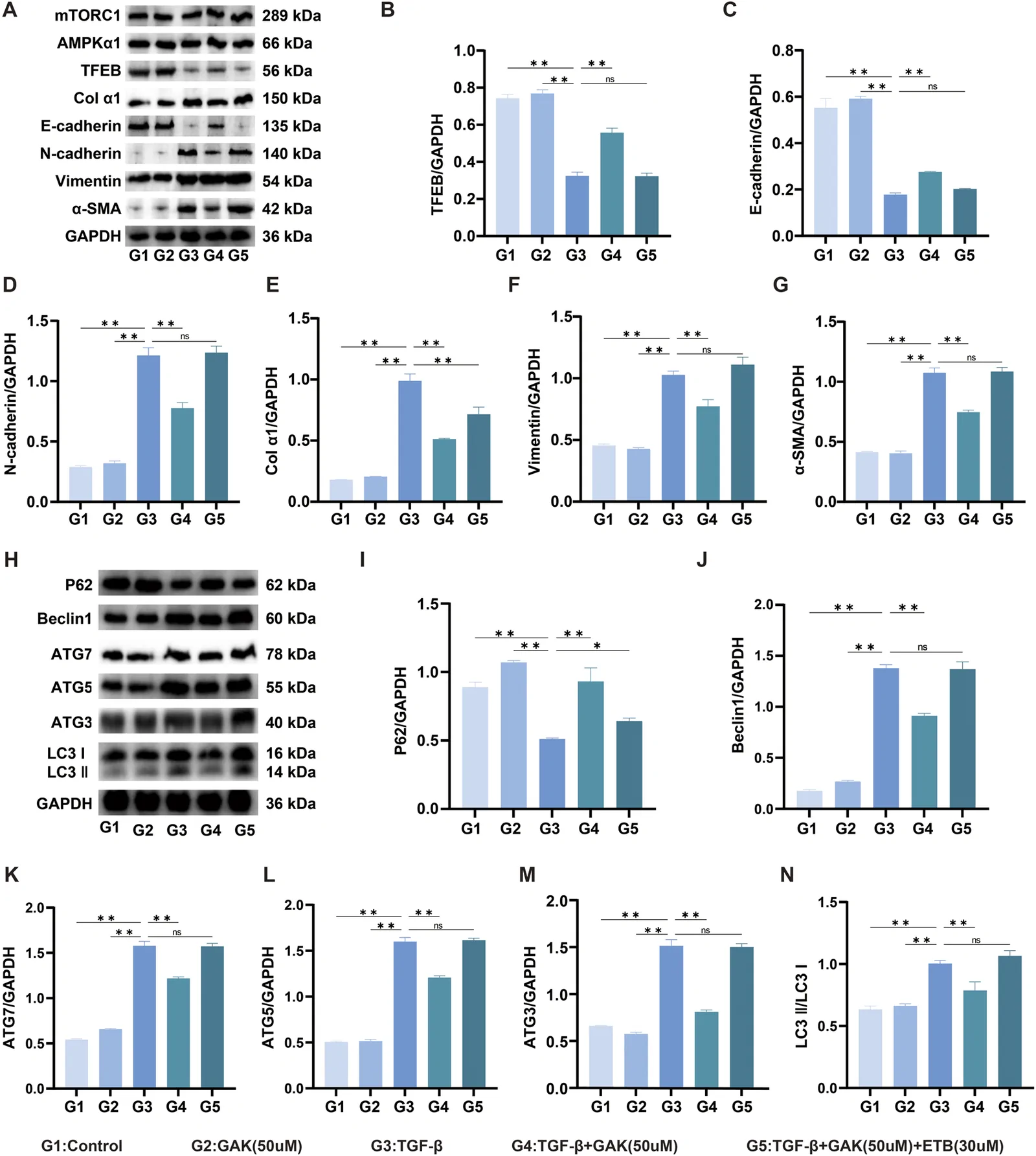
TFEB inhibition attenuates the protective effects of GAK on EMT, extracellular matrix accumulation, and autophagy regulation. HK-2 cells were treated with TGF-β1 and GAK in the presence or absence of the TFEB inhibitor eltrombopag (ETB, 30 μM), as indicated. **(A)** Representative Western blot images of TFEB and EMT- or fibrosis-related proteins, including E-cadherin, N-cadherin, Col α1, vimentin, and α-SMA, with GAPDH as the loading control. **(B–G)** Densitometric quantification of TFEB/GAPDH **(B)**, E-cadherin/GAPDH **(C)**, N-cadherin/GAPDH **(D)**, Col α1/GAPDH **(E)**, vimentin/GAPDH **(F)**, and α-SMA/GAPDH **(G)**. **(H)** Representative Western blot images of autophagy-related proteins, including P62, Beclin1, ATG7, ATG5, ATG3, and LC3. **(I–N)** Densitometric quantification of P62/GAPDH **(I)**, Beclin1/GAPDH **(J)**, ATG7/GAPDH **(K)**, ATG5/GAPDH **(L)**, ATG3/GAPDH **(M)**, and the LC3-II/LC3-I ratio **(N)**. Data are presented as mean ± SD. Statistical significance was determined by one-way ANOVA followed by Tukey’s multiple-comparisons test. Ns, not significant; *P < 0.05; **P < 0.01.

Previous studies suggest that TFEB is a key regulator of lysosomal biogenesis and autophagy (Ren et al., 2024; Alesi et al., 2024; Du et al., 2024). Therefore, we next examined whether TFEB is involved in the effects of GAK. Initial analysis showed that TGF-β1 reduced TFEB expression, which indicates that TFEB may be suppressed during TGF-β1 induced fibrotic stress (Figures 5G,H; Supplementary Figure S1F). In follow-up experiments, we found that GAK significantly increased TFEB levels compared with the TGF-β1 group, which suggests that GAK may restore TFEB expression under fibrotic conditions. To test the functional role of TFEB, we added EBT, a TFEB inhibitor, to the TGF-β1 + GAK treatment. TFEB inhibition weakened the protective effects of GAK on EMT and fibrotic markers. This was shown by lower E-cadherin and higher N-cadherin, α-SMA, vimentin, and Col α1 compared with the TGF-β1 + GAK group (Figures 6A–G; Supplementary Figure S5). In parallel, TFEB inhibition partly reversed the GAK-related changes in autophagy proteins (P62, Beclin1, ATG7, ATG5, ATG3, and the LC3II/LC3I ratio) (Figures 6H–N; Figure). However, when we further examine the upstream genes of TFEB, such as AMPKα1 and mTORC1, similar exchanging trend of these two genes were not observed, indicating the target of GAK was TFEB rather than its upstream genes (Figure 6A).

### 3.6 GAK ameliorates UUO induced renal fibrosis in mice

**FIGURE 7:**
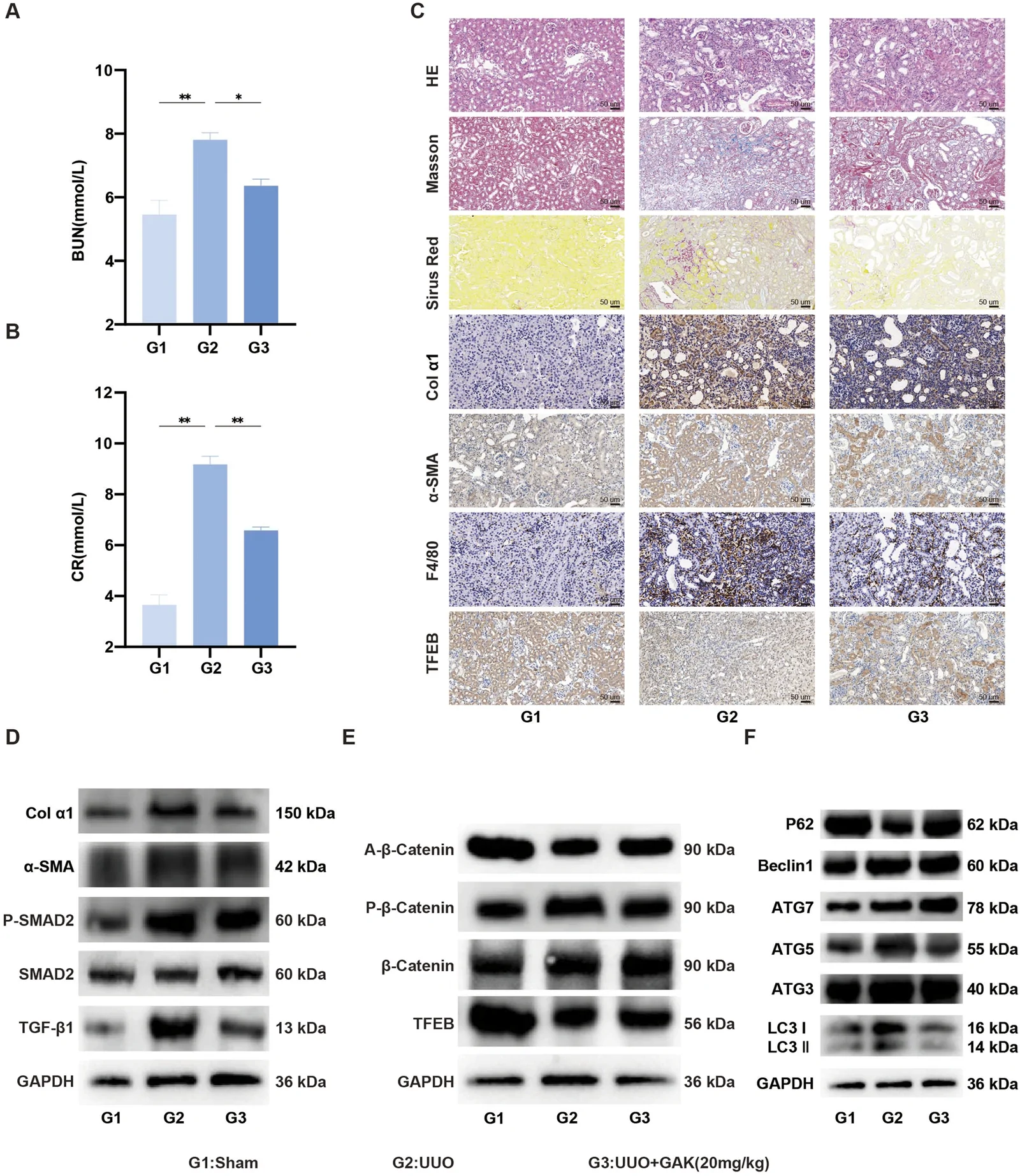
GAK alleviates UUO-induced renal fibrosis and modulates profibrotic signaling and TFEB-related autophagy *in vivo*. Male C57BL/6 mice were assigned to the Sham (n = 6), UUO (n = 6), and UUO + GAK (n = 6) groups. GAK was administered at 20 mg/kg for 14 days after UUO surgery. **(A,B)** Serum blood urea nitrogen (BUN) **(A)** and serum creatinine **(B)** levels in the indicated groups. **(C)** Representative renal histology and immunostaining images of H&E staining, Masson’s trichrome staining, Sirius Red and immunostaining for α-SMA, TFEB, F4/80 and Col α1 staining, scale bars are indicated in the images. **(D)** Representative Western blot images of fibrosis- and TGF-β1/SMAD2-related proteins, including Col α1, α-SMA, p-SMAD2, total SMAD2, and TGF-β1, in kidney tissues, with GAPDH as the loading control. **(E)** Representative Western blot images of active β-catenin, phospho-β-catenin, total β-catenin, and TFEB in kidney tissues, with GAPDH as the loading control. **(F)** Representative Western blot images and quantification of autophagy-related proteins, including P62, Beclin1, ATG7, ATG5, ATG3, and LC3, in kidney tissues, with GAPDH as the loading control. Data are presented as mean ± SD. Statistical significance was determined by one-way ANOVA followed by Tukey’s multiple-comparisons test. Ns, not significant; *P < 0.05; **P < 0.01.

To determine whether the molecular changes observed in TGF-β1-stimulated HK-2 cells were reproduced *in vivo*, we examined the effect of GAK in the UUO mouse model. Compared with the Sham group, UUO markedly increased serum BUN and CR levels, whereas GAK treatment significantly reduced these increases (Figures 7A,B). Histological analysis showed evident tubular injury and interstitial fibrosis in UUO kidneys. Masson’s trichrome and Sirius Red staining revealed increased collagen deposition after UUO, while GAK treatment attenuated these pathological changes. Consistently, IHC staining showed that UUO increased Col α1 and α-SMA expression, indicating enhanced ECM accumulation and myofibroblast activation; these effects were reduced by GAK. F4/80 staining further showed that GAK decreased UUO induced macrophage infiltration, and TFEB staining was partially restored in GAK treated kidneys (Figure 7C).

RT-qPCR analysis of Col α1, α-SMA, TFEB, P62 Beclin1, ATG7, ATG5 and ATG3 in UUO kidney tissues confirmed same changing trend with TGF-β stimulated HK-2 cells, and GAK could partially reverse these changes (Supplementary Figure S7). Western blot analysis also further confirmed that the *in vivo* molecular pattern was consistent with the HK-2 cell findings. UUO increased Col α1, α-SMA, TGF-β1, and p-SMAD2 levels, whereas GAK reduced these fibrotic and TGF-β1/SMAD2 related changes (Figure 7D; Supplementary Figure S8). GAK also partly reversed UUO induced alterations in β-catenin pathway related proteins and restored TFEB expression (Figure 7E; Supplementary Figure S8). In addition, UUO caused dysregulation of autophagy-related proteins, including P62, Beclin1, ATG7, ATG5, ATG3, and LC3B-I/II, while GAK partly normalized these changes (Figure 7F; Supplementary Figure S8).

## 4 Discussion

In this study, we propose a pharmacology-to-disease framework in which GAK attenuates renal fibrotic responses in association with reduced profibrotic signaling and improved lysosome-autophagy function. In TGF-β1–stimulated HK-2 cells, GAK reduced EMT and ECM accumulation. GAK increased E-cadherin and decreased α-SMA, vimentin, Col αI, and fibronectin. These changes occurred with lower canonical TGF-β1 signaling activity, shown by reduced p-SMAD2, and with less β-catenin nuclear redistribution. Pathway inhibitor experiments suggested that these protective effects are partly linked to the TGF-β1/SMAD2 and Wnt/β-catenin pathways. We also found that GAK improved autophagy balance and autophagic flux, supported by tandem fluorescent LC3 and ultrastructural analyses, and this was accompanied by increased TFEB signaling. In the UUO model, GAK reduced histologic fibrosis and reproduced the main molecular changes seen *in vitro*. Overall, our results support current models that view kidney fibrosis as a network disease driven by coupled signaling (TGF-β1/SMAD2 and Wnt/β-catenin) and maladaptive stress programs that sustain epithelial injury and ECM overproduction (Ren et al., 2024; Dai et al., 2022; Ruby et al., 2023; Jiang et al., 2025; Li et al., 2024; Yu et al., 2022; Yin et al., 2026).

This potential dual effect is consistent with previous reports on GAK. Recent studies describe GAK as a methoxylated flavone with anti-inflammatory and antioxidant effects in several disease models (El Menyiy et al., 2023). Other studies suggest that GAK reduces key inflammatory pathways, such as NF-κB and JAK/STAT, and lowers inflammatory mediators, such as TNF-α and IL-6 (Lu et al., 2025). This matters because inflammation can start and strengthen profibrotic signaling during chronic kidney injury. In renal fibrosis, inflammation and oxidative stress can activate TGF-β1, make epithelial cells more prone to EMT, and disrupt mitochondrial and lysosomal function. These changes can weaken protein control and reduce lysosome-based clearance (Ren et al., 2024; Ruby et al., 2023; Jiang et al., 2025; Li et al., 2024). Based on these findings, we propose a simple drug–disease link: GAK may reduce the background injury signals that keep TGF-β1 and Wnt pathways active, and it may also improve intracellular quality control. This view fits a common idea in natural-product antifibrotic research. Many flavones may not act on one target only. Instead, they may produce benefit by making moderate changes at several points, including cytokine output, redox balance, and stress pathways linked to TGF-β1 and Wnt signaling, which helps move the system away from a persistent profibrotic state (Jiang et al., 2025).

We interpret the inhibition of TGF-β1/SMAD2 and Wnt/β-catenin signaling in the context of their known crosstalk in renal fibrosis. Canonical TGF-β1/SMAD signaling is a major driver of tubulointerstitial fibrosis and promotes EMT-related gene programs and ECM production (Ruby et al., 2023; Jiang et al., 2025). However, recent reviews note that SMAD family members can have different roles depending on the setting. For this reason, changes in p-SMAD2 mainly indicate the activity level of canonical TGF-β1 signaling, and they do not prove that SMAD2 is the main profibrotic effector in all cases (Yin et al., 2026). Wnt/β-catenin signaling is also often reactivated after adult kidney injury, and it can promote epithelial plasticity and fibrotic remodeling (Li et al., 2021b; Adeerjiang et al., 2025). Recent studies show that post-translational changes can control β-catenin stability and its ability to drive transcription, and these changes can affect fibrosis severity (Gu et al., 2023; Cohen et al., 2024). In addition, Wnt signaling shapes the fibrotic niche through cell–cell interactions. Inflammatory fibroblasts and certain macrophage subsets can reinforce each other through Wnt/β-catenin–dependent signaling, which supports fibrotic conversion and ECM accumulation (Cohen et al., 2024). This means that β-catenin activation is not only a cell-intrinsic marker. It may also reflect broader pathological communication between cell types. Against this background, our finding that GAK reduces β-catenin activation and nuclear redistribution, together with reduced TGF-β1 pathway activity, supports a model in which GAK acts on a connected TGF-β1–Wnt network rather than on one isolated pathway (Gu et al., 2023; Li et al., 2021b; Jiang et al., 2025; Yin et al., 2026; Adeerjiang et al., 2025; Cohen et al., 2024; Somanader et al., 2024; Naillat et al., 2024). Early clinical data also show that blocking Wnt secretion can suppress Wnt signaling in humans (Zhou et al., 2025).

A key contribution of our study is that we connect TFEB-related autophagy function to the antifibrotic effects of GAK. Autophagy in renal fibrosis depends on context. Differences across studies often reflect differences in cell type and disease stage, and they also depend on whether autophagic flux is intact or blocked at the lysosome (Dai et al., 2022; Ruby et al., 2023; Zhang et al., 2024). For this reason, many studies now separate autophagosome formation from autophagosome clearance (fusion and degradation). Lysosome dysfunction can cause LC3-positive vesicles to build up, which may look like autophagy activation but actually reflects impaired clearance (Dai et al., 2022; Ruby et al., 2023). TFEB regulates lysosome biogenesis and many autophagy-related genes. Recent studies show that reduced TFEB activity is linked to impaired fusion, abnormal tubular cell-cycle control, and progressive fibrosis. In contrast, restoring TFEB activity, often through mTORC1 regulation, can improve fibrosis in UUO and diabetic kidney disease models (Ren et al., 2024; Du et al., 2024; Yang et al., 2025; Ai et al., 2025). In our study, GAK increased TFEB signaling and improved flux-based readouts. TFEB inhibition also partly weakened GAK’s protective effects. These results support an association among TFEB activity, improved autophagic flux, and antifibrotic protection. However, TFEB is not always protective. Some studies report that lowering TFEB or autophagy activity can reduce fibrosis in certain settings (Lee et al., 2022). Other work shows that TFEB can contribute to disease in specific genetic kidney disorders (Alesi et al., 2024). Based on our data, we propose a testable model. GAK may rebalance a connected system by reducing profibrotic signaling (TGF-β1/SMAD2 and β-catenin) and improving TFEB-driven lysosome function. This combination may help restore more effective autophagic processing and support epithelial stability. This model also fits reports that stronger autophagic degradation of mature TGF-β1 can reduce renal fibrosis (Li et al., 2025). It also fits proposed β-catenin-based links between injury signaling and autophagy programs (Li et al., 2026).

In summary, our study highlights two mechanistic advances with potential translational value. First, we identify GAK as a bioactive flavone that reduces renal fibrotic phenotypes in both cellular and UUO models. We link this effect to coordinated suppression of two major profibrotic pathways, TGF-β1/SMAD2 and Wnt/β-catenin, instead of a single pathway. Second, we include TFEB-related lysosome–autophagy function in the mechanistic model and show that GAK improves autophagic flux. This flux improvement may help connect drug activity to more sustained suppression of profibrotic signaling (Ren et al., 2024; Li et al., 2026; Dai et al., 2022; Ruby et al., 2023). Together, these findings support a dual approach: reducing profibrotic gene programs while improving degradative capacity to limit ongoing epithelial stress and maladaptive repair.

Several limitations should be acknowledged. First, although the present study supports an association between GAK treatment, reduced profibrotic signaling, and restoration of TFEB-related autophagy-lysosome regulation, the direct molecular target of GAK remains unidentified. Future studies using target-identification approaches, such as chemical proteomics, CETSA, pull-down assays, or binding validation, will be needed to distinguish primary targets from downstream pathway changes (Ren et al., 2024; Li et al., 2026; Du et al., 2024; Yang et al., 2025). Second, while pharmacological intervention and the current rescue experiments support the involvement of autophagy and profibrotic signaling pathways, additional genetic gain- and loss-of-function studies targeting TFEB, β-catenin, and SMAD2 are still required to establish a more definitive causal mechanism (Cohen et al., 2024).Third, the UUO model is a widely used model of renal fibrosis but does not fully recapitulate all forms of chronic kidney disease, and renal functional indices such as BUN and serum creatinine may be relatively insensitive in this unilateral model. Fourth, pharmacokinetic properties, long term toxicity, dose response safety, and long-term therapeutic efficacy of GAK have not yet been systematically evaluated. Future studies using additional kidney disease models and direct target validation strategies will be important to further assess the therapeutic potential of GAK.

## 5 Conclusion

In conclusion, our study demonstrates that GAK attenuates renal fibrotic responses in both TGF-β1-stimulated HK-2 cells and the UUO mouse model. *In vitro*, GAK reduced EMT and ECM accumulation, as reflected by increased E-cadherin expression and decreased expression of α-SMA, vimentin, Col α1, and fibronectin. *In vivo*, GAK alleviated UUO-induced renal histological injury, collagen deposition, and fibrosis-related marker expression. Mechanistically, these protective effects were associated with reduced activation of TGF-β1/SMAD2 and β-catenin signaling, together with improvement of TFEB-related autophagy-lysosome regulation and autophagic flux. Consent for publication: Not Applicable.

## Data Availability

The original contributions presented in the study are included in the article/Supplementary Material, further inquiries can be directed to the corresponding authors.

## Supplementary material

SUPPLEMENTARY FIGURE S1 GAK regulates autophagy-related and TFEB mRNA expression in TGF-β1-stimulated HK-2 cells. HK-2 cells were stimulated with TGF-β1 in the presence or absence of GAK, as indicated. RT-qPCR analysis was performed to measure the relative mRNA expression of P62 **(A)**, Beclin1 **(B)**, ATG7 **(C)**, ATG5 **(D)**, ATG3 **(E)**, and TFEB **(F)**. Gene expression was normalized to the indicated internal control and expressed relative to the control group. Data are presented as mean ± SD. Statistical significance was determined by one-way ANOVA followed by Tukey’s multiple-comparisons test. Ns, not significant; *P < 0.05; **P < 0.01.

SUPPLEMENTARY FIGURE S2 Quantification of autophagy-related proteins in TGF-β1-stimulated HK-2 cells treated with GAK. Densitometric quantification of P62 /GAPDH **(A)**, Beclin 1/GAPDH **(B)**, ATG7/GAPDH **(C)**, ATG5/GAPDH **(D)**, ATG3/GAPDH **(E)**, and the LC3-II/LC3-I ratio **(F)** corresponding to the Western blot data shown in Figure 5A. Data are presented as mean ± SD. Statistical significance was determined by one-way ANOVA followed by Tukey’s multiple-comparisons test. Ns, not significant; *P < 0.05; **P < 0.01.

SUPPLEMENTARY FIGURE S3 Quantification of P62 and LC3 immunofluorescence signals in HK-2 cells. Quantification of P62 fluorescence intensity **(A)** and LC3 fluorescence intensity or puncta signal **(B)** in HK-2 cells treated with TGF-β1 in the presence or absence of GAK, as indicated. Fluorescence intensity was quantified from representative fields obtained from three independent biological experiments. Data are presented as mean ± SD. Statistical significance was determined by one-way ANOVA followed by Tukey’s multiple-comparisons test. Ns, not significant; *P < 0.05; **P < 0.01.

SUPPLEMENTARY FIGURE S4 Bafilomycin A1 treatment and TFEB knockdown attenuate the protective effects of GAK in TGF-β1-stimulated HK-2 cells. **(A)** Representative Western blot analysis of fibrosis and EMT related proteins, including Col α1, E-cadherin, N-cadherin, vimentin, and α-SMA, as well as autophagy-related proteins P62, LC3B-I/II, and TFEB in HK-2 cells treated with TGF-β1, TGF-β1 + GAK, or TGF-β1 + GAK + bafilomycin A1 (Baf1). HK-2 cells were stimulated with TGF-β1 (10 ng/mL) and treated with GAK (50 μM) for 48 h BafA1 (20 nM) was added for the final 12 h before protein collection to block lysosomal degradation. GAPDH was used as the loading control. **(B)** Representative Western blot analysis of Col α1, E-cadherin, N-cadherin, vimentin, α-SMA, P62, LC3B-I/II, and TFEB in TGF-β1-stimulated HK-2 cells after TFEB knockdown. Cells were transfected with TFEB siRNA or negative control siRNA and then treated with TGF-β1 with or without GAK, as indicated. GAPDH was used as the loading control. Data are representative of three independent experiments.

SUPPLEMENTARY FIGURE S5 TFEB inhibition attenuates the effects of GAK on EMT- and fibrosis-related mRNA expression. HK-2 cells were stimulated with TGF-β1 and treated with GAK in the presence or absence of ETB, as indicated. RT-qPCR analysis was performed to measure the relative mRNA expression of TFEB **(A)**, E-cadherin **(B)**, N-cadherin **(C)**, Col α1 **(D)**, vimentin **(E)**, and α-SMA **(F)**. Gene expression was normalized to the indicated internal control and expressed relative to the control group. Data are presented as mean ± SD. Statistical significance was determined by one-way ANOVA followed by Tukey’s multiple-comparisons test. Ns, not significant; *P < 0.05; **P < 0.01.

SUPPLEMENTARY FIGURE S6 TFEB inhibition modifies the effects of GAK on autophagy-related mRNA expression. HK-2 cells were stimulated with TGF-β1 and treated with GAK in the presence or absence of ETB, as indicated. RT-qPCR analysis was performed to measure the relative mRNA expression of P62 **(A)**, Beclin 1 **(B)**, ATG7 **(C)**, ATG5 **(D)**, and ATG3 **(E)**. Gene expression was normalized to the indicated internal control and expressed relative to the control group. Data are presented as mean ± SD. Statistical significance was determined by one-way ANOVA followed by Tukey’s multiple-comparisons test. Ns, not significant; *P < 0.05; **P < 0.01.

SUPPLEMENTARY FIGURE S7 GAK regulates autophagy-related mRNA expression in UUO mouse kidneys. Male C57BL/6 mice were assigned to the Sham (n = 6), UUO (n = 6), and UUO + GAK (n = 6) groups. GAK was administered at 20 mg/kg for 14 days after UUO surgery. **(A-H)** RT-qPCR analysis of Col α1 **(A)**, α-SMA **(B)**, TFEB **(C)**, P62 **(D)**, Beclin1 **(E)**, ATG7 **(F)**, ATG5 **(G)** and ATG3 **(H)** mRNA expression in kidney tissues. Data are presented as mean ± SD. Statistical significance was determined by one-way ANOVA followed by Tukey’s multiple-comparisons test. Ns, not significant; *P < 0.05; **P < 0.01.

SUPPLEMENTARY FIGURE S8 GAK regulates autophagy-related protrin expression in UUO mouse kidneys. Male C57BL/6 mice were assigned to the Sham (n = 6), UUO (n = 6), and UUO + GAK (n = 6) groups. GAK was administered at 20 mg/kg for 14 days after UUO surgery. **(A-O)** Densitometric quantification of Col α1/GAPDH **(B)**, α-SMA/GAPDH **(C)**, p-SMAD2/GAPDH **(D)**, and SMAD2/GAPDH **(E)**, Beclin1/GAPDH **(F)**, ATG7/GAPDH **(G)**, ATG5/GAPDH **(H)**, ATG3/GAPDH **(I)**, LC-II/LC-I, active β-catenin/GAPDH **(K)**, phospho-β-catenin/GAPDH **(L)**, and total β-catenin/GAPDH **(M)**, TGF-β **(N)** and TFEB **(O)** in kidney tissues from the Sham, UUO, and UUO + GAK groups. Data are presented as mean ± SD. Statistical significance was determined by one-way ANOVA followed by Tukey’s multiple-comparisons test. Ns, not significant; *P < 0.05; **P < 0.01.
